# Glucosylsphingosine Causes Hematological and Visceral Changes in Mice—Evidence for a Pathophysiological Role in Gaucher Disease

**DOI:** 10.3390/ijms18102192

**Published:** 2017-10-20

**Authors:** Jan Lukas, Claudia Cozma, Fan Yang, Guido Kramp, Anja Meyer, Anna-Maria Neßlauer, Sabrina Eichler, Tobias Böttcher, Martin Witt, Anja U. Bräuer, Peter Kropp, Arndt Rolfs

**Affiliations:** 1 Albrecht-Kossel Institute of the Rostock University Medical Center, 18147 Rostock, Germany; fan.yang@med.uni-rostock.de (F.Y.); boettcherT@dbknb.de (T.B.); arndt.rolfs@med.uni-rostock.de (A.R.); 2 Centogene AG, 18057 Rostock, Germany; claudia.cozma@centogene.com (C.C.); guido.kramp@centogene.com (G.K.); sabrina.eichler@centogene.com (S.E.); 3 Department of Anatomy, Rostock University Medical Center, 18057 Rostock, Germany; anja.meyer@med.uni-rostock.de (A.M.); anna-maria.nesslauer@med.uni-rostock.de (A.-M.N.); martin.witt@med.uni-rostock.de (M.W.); anja.braeuer@med.uni-rostock.de (A.U.B.); 4 Institute for Medical Psychology and Medical Sociology, Rostock University Medical Center, 18147 Rostock, Germany; peter.kropp@med.uni-rostock.de

**Keywords:** sphingolipids, glucocerebrosidase, biomarker, storage disease pathology, chemically-induced phenotype

## Abstract

Glucosylceramide and glucosylsphingosine are the two major storage products in Gaucher disease (GD), an inherited metabolic disorder caused by a deficiency of the lysosomal enzyme glucocerebrosidase. The build-up of glucosylceramide in the endoplasmic reticulum and prominent accumulation in cell lysosomes of tissue macrophages results in decreased blood cell and platelet counts, and skeletal abnormalities. The pathological role of the deacylated form of glucosylceramide, glucosylsphingosine (lyso-Gb1), a recently identified sensitive and specific biomarker for GD, is not well investigated. We established a long-term infusion model in C57BL/6JRj mice to examine the effect of lyso-Gb1 on representative hallmark parameters of GD. Mice received lyso-Gb1 at a dosage of 10 mg·kg^−1^ per day as a continuous subcutaneous administration, and were routinely checked for blood lyso-Gb1 levels using liquid chromatography-multiple reaction monitoring mass spectrometry (LC/MRM-MS) measurements at four-weekly intervals throughout treatment. The C57BL/6JRj mice showed a stable increase of lyso-Gb1 up to->500-fold greater than the normal reflecting concentrations seen in moderately to severely affected patients. Furthermore, lyso-Gb1 accumulated in peripheral tissues. The mice developed hematological symptoms such as reduced hemoglobin and hematocrit, increased spleen weights and a slight inflammatory tissue response after eight weeks of treatment. The above findings indicate a measurable visceral and hematological response in treated mice that suggests a role for lyso-Gb1 in the development of peripheral signs of GD.

## 1. Introduction

Biomarkers in lysosomal storage diseases (LSD) are employed to augment primary disease diagnosis and monitoring of disease progression, and therapeutic efficacy. Gaucher disease (GD) is a particular LSD caused by a deficiency of glucocerebrosidase (GCase), and several biomarkers have been identified. Chitotriosidase activity was first introduced as a biomarker in 1994 [1] and has since proven to be particularly useful for both diagnosis and monitoring in patients with GD type 1. However, its use is precluded in patients harboring a common null allele in the CHIT1 gene [2]. The chemokine CCL18 was demonstrated to act as a surrogate marker produced by Gaucher cells, and has also been described as a reliable biomarker [3]. Both of these biomarkers can easily be assessed in patient plasma samples. However, they have not yet been linked to disease pathology specifically, and can only be regarded as reflecting a secondary abnormality.

The accumulation of the glycosphingoid base, glucosylsphingosine (lyso-Gb1), in GD patients has previously been documented [4]. Lyso-Gb1 is an amphipathic compound that has been reported to originate from the enzymatic action of lysosomal acid ceramidase on the cell’s primary glycosphingolipid storage product, glucosylceramide [5,6]. Lyso-Gb1 is highly abundant in the brain tissue of patients with neuronopathic GD, but not in non-neuronopathic GD patients [7]. This suggests a role in the neuropathology of acute and subacute GD type 2 and 3. Moreover, lyso-Gb1 is strongly elevated in the plasma of all GD patients, and it can therefore be speculated that it is closely linked to disease pathology.

Recent methodological advances have allowed the relatively simple quantitative detection of lyso-Gb1 using liquid chromatography-tandem mass spectrometry (LC–MS/MS) in patient plasma samples. Previous studies have subsequently established lyso-Gb1 as a sensitive and specific biomarker for the diagnosis of GD in many laboratories [8,9].

Many pathophysiological roles have been attributed to sphingolipids [10], lyso-sphingolipids [11], and in particular, lyso-Gb1 [12]. Schueler and colleagues demonstrated a toxic effect on cultured neuronal cells in the presence of a functional GCase enzyme, indicating that lyso-Gb1 was readily taken up by the cells, likely via endocytosis, and redistributed to the lysosomes. Animal models of GD display a broad spectrum of neurological, hematological and visceral phenotypes based on the nature of the genetic defect. Both genetic and non-genetic (chemically-induced) GD mouse models provide informative insights into the disease pathology, often closely resembling the human phenotype (for an extensive review see [13]). However, none of the disease models developed so far permit an isolated analysis of the effects attributable to sphingolipid storage alone. The objective of the present study was to examine the pathogenic role of lyso-Gb1 in visceral disease features after continuous systemic subcutaneous administration in genetically normal C57BL/6JRj mice.

## 2. Results

### 2.1. Subcutaneously Administered Lyso-Gb1 Can Be Detected in Blood and Organs

**Figure 1:**
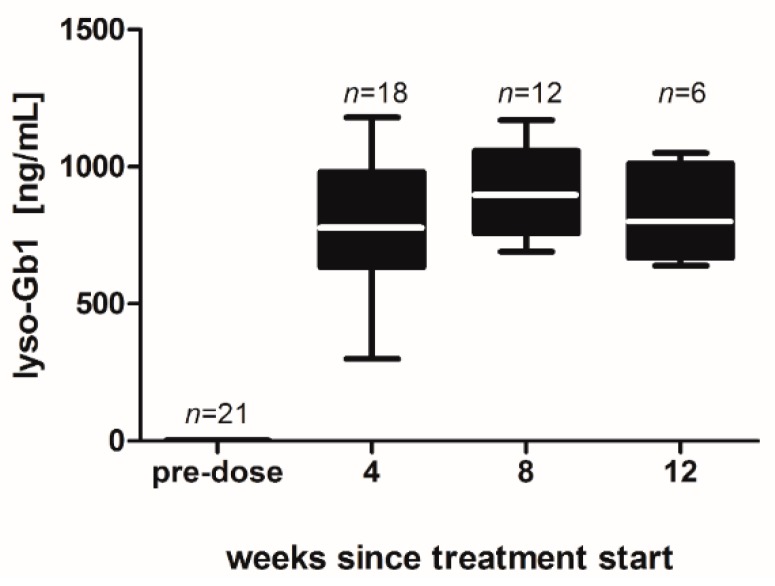
Blood lyso-Gb1 level in treated mice over time. Mice received subcutaneous lyso-Gb1 for 12 weeks using osmotic mini pumps. Lyso-Gb1 values were increased at first sampling after four weeks, and throughout the treatment phase thereafter. Vehicle-treated (or pre-dose) animals had lower, yet detectable lyso-Gb1 values of 1.2–1.5 ng/mL blood. The number of animals tested is indicated in the figure for each data point. The boxes indicate the median and 25th and 75th percentiles, the whiskers of the graph show the minimum and maximum values.

Male C57BL/6JRj mice were equipped with subcutaneous osmotic mini pumps in the back of their necks to achieve long-term administration of lyso-Gb1. Recurrent dried blood spot (DBS) sampling was carried out in order to investigate the development of lyso-Gb1 levels in the mouse organ system. Monitoring revealed that lyso-Gb1 levels were strongly elevated after four, eight and 12 weeks, lyso-Gb1 levels ranging between 700 and 900 ng/mL. This represented a >500-fold increase compared with vehicle-treated mice (Figure 1) and represents concentrations seen in moderately to severely affected untreated GD patients [9].

**Figure 2:**
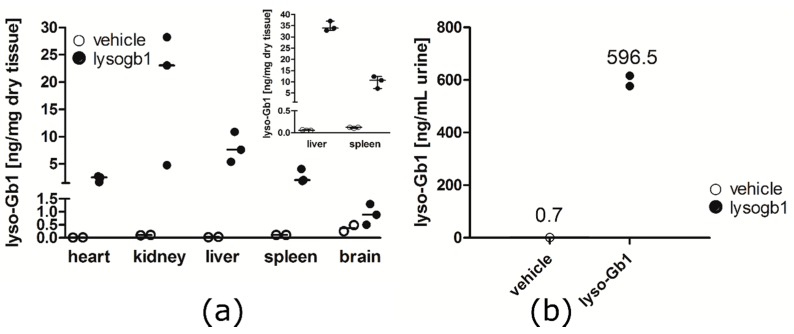
Determination of organ lyso-Gb1 content. Animals subcutaneously treated for four weeks with 10 mg·kg^−1^ per day were sacrificed, and the heart, kidney, liver, spleen and brain were analyzed for lyso-Gb1 accumulation. (**a**) All peripheral organs of the treated mice showed strongly elevated levels of lyso-Gb1 compared to control animals. The inlayed diagram shows the results for the liver and spleen of previously phosphate-buffered saline perfused mice. Lyso-Gb1-treated animals are shown as closed circles, vehicle-treated animals as open circles. Each organ sample was measured in duplicate. The horizontal lines indicate the median values for each group; (**b**) Urine was collected for 24 h from one group of four (untreated) and two groups of three (treated) animals per cage at six weeks after treatment start. The two values for the treated groups are indicated as aligned dots. The obtained mean value for lyso-Gb1 is indicated above. Lyso-Gb1 content in the treated animals refer to a calculated excretion rate of 0.6 ng·min^−1^.

After four weeks from treatment start the organs of the mice were subjected to lyso-Gb1 analysis. Median basal levels of lyso-Gb1 were 0.015 (heart), 0.105 (kidney), 0.029 (liver), 0.110 (spleen) and 0.370 (brain) ng/mg dry tissue (Figure 2a). The treated animals showed the highest levels of lyso-Gb1 in the kidney, indicating at least partial urinary elimination of the amphipathic lyso-Gb1. This was verified by the urinary levels of lyso-Gb1 (Figure 2b). A dramatic increase in lyso-Gb1 levels was observed in all other peripheral organs, where it was more pronounced in the liver compared to, for example, the spleen, where it was less marked. A small, two-fold increase of brain lyso-Gb1 was attributed to the carryover of blood in the tissue capillaries, which had not been cleared by perfusion prior to analysis. In a subsequent experiment, the mice were perfused using phosphate-buffered saline prior to euthanasia and the liver and spleen showed lyso-Gb1 levels in the same range (inlay in Figure 2a), which suggests organ uptake of the lyso-Gb1 probe.

### 2.2. Abnormal Blood Parameters after Administration of Lyso-Gb1

**Figure 3:**
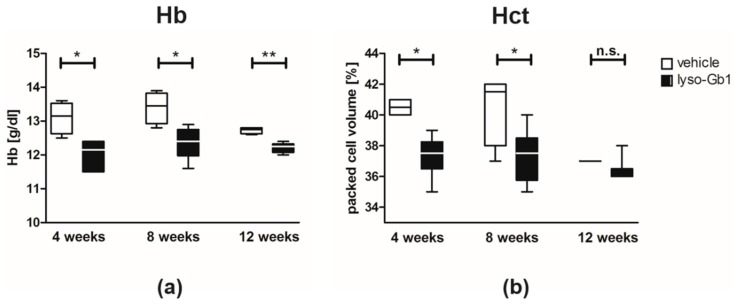
Blood parameter changes in lyso-Gb1 treated C57BL/6JRj mice. (**a**) Hb values were below the baseline value at each time point, indicating mild anemia; (**b**) Hct values significantly differed at four and eight weeks after treatment initiation. Values were derived from four control and six lyso-Gb1 treated mice at each indicated time point. The black and white boxes indicate the median and 25th and 75th percentiles, the whiskers of the graph show the minimum and maximum values. Differences between treatment groups at each time point were analyzed using the two-tailed Mann–Whitney test. Results were considered to be statistically significant for * *p* < 0.05, ** *p* < 0.01, n.s.: not significant.

Blood count analyses were performed to assess whether the high lyso-Gb1 levels caused systemic damage. The animals exhibited a state of slightly reduced Hb (Figure 3a) at a range which had previously been recognized as a mild anemia [14]. In addition, the animals displayed lowered Hct (Figure 3b). There appeared to be a reduction in Hct values in the control animals at week 12. Even though this was not statistically significant we cannot entirely exclude the possibility that this reduction could be a consequence of exposure to the used vehicle (50% dimethylsulfoxid (DMSO)/50% propylene glycol), as propylene glycol has previously been shown to exert some hematological effects (www.atsdr.cdc.gov). The low infusion volume, and reported tolerability of the compound in markedly higher doses [15], makes this unlikely. More importantly, however, is the fact that there was no further progression in the Hct reduction in the lyso-Gb1 treated animals. There was no thrombocytopenia or abnormalities in white blood cells detectable within the mice. Table S1A,B summarize all relevant parameters compiled in this study.

### 2.3. Lyso-Gb1 Induces a Visceral Inflammatory Response in Mice

**Figure 4:**
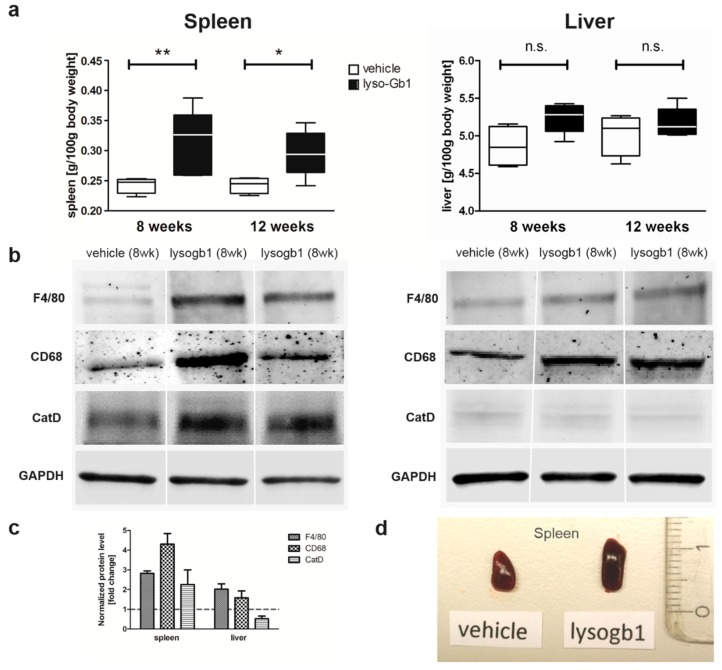
Spleen and liver abnormalities of lyso-Gb1 treated C57BL/6JRj mice. (**a**) The spleen of treated mice was enlarged versus controls eight and 12 weeks after treatment initiation. Liver weight was not statistically significantly different compared with controls. Values were derived from four control and six lyso-Gb1-treated mice at each time point. The black and white boxes indicate the median and 25th and 75th percentiles, the whiskers of the graph show the minimum and maximum values. Differences between treatment groups at each time point were analyzed using the two-tailed Mann-Whitney test and considered statistically significant for * *p* < 0.05, ** *p* < 0.01; (**b**) Western blot analysis of inflammatory tissue markers F4/80 and CD68. A pronounced increase in spleen levels for F4/80 and CD68 was observed in the lyso-Gb1 treated mice after eight weeks. A similar increase was noted in the livers of the same animals. Cathepsin D (CatD) was elevated in the spleen, but no change was observed in the liver; (**c**) Quantification of the Western blot. Spleen and liver of eight weeks treated mice were subjected to Western blot analysis. Each group is represented by three different animals. All samples were tested twice. The signal strength of F4/80, CD68 and CatD was determined in relation to Glyceraldehyde-3-phosphate dehydrogenase (GAPDH) and presented as a fold change of vehicle control; (**d**) Comparison of half a spleen from a control and lyso-Gb1 treated animals. The organ samples illustrate the change in size and a distinctive color.

**Figure 5:**
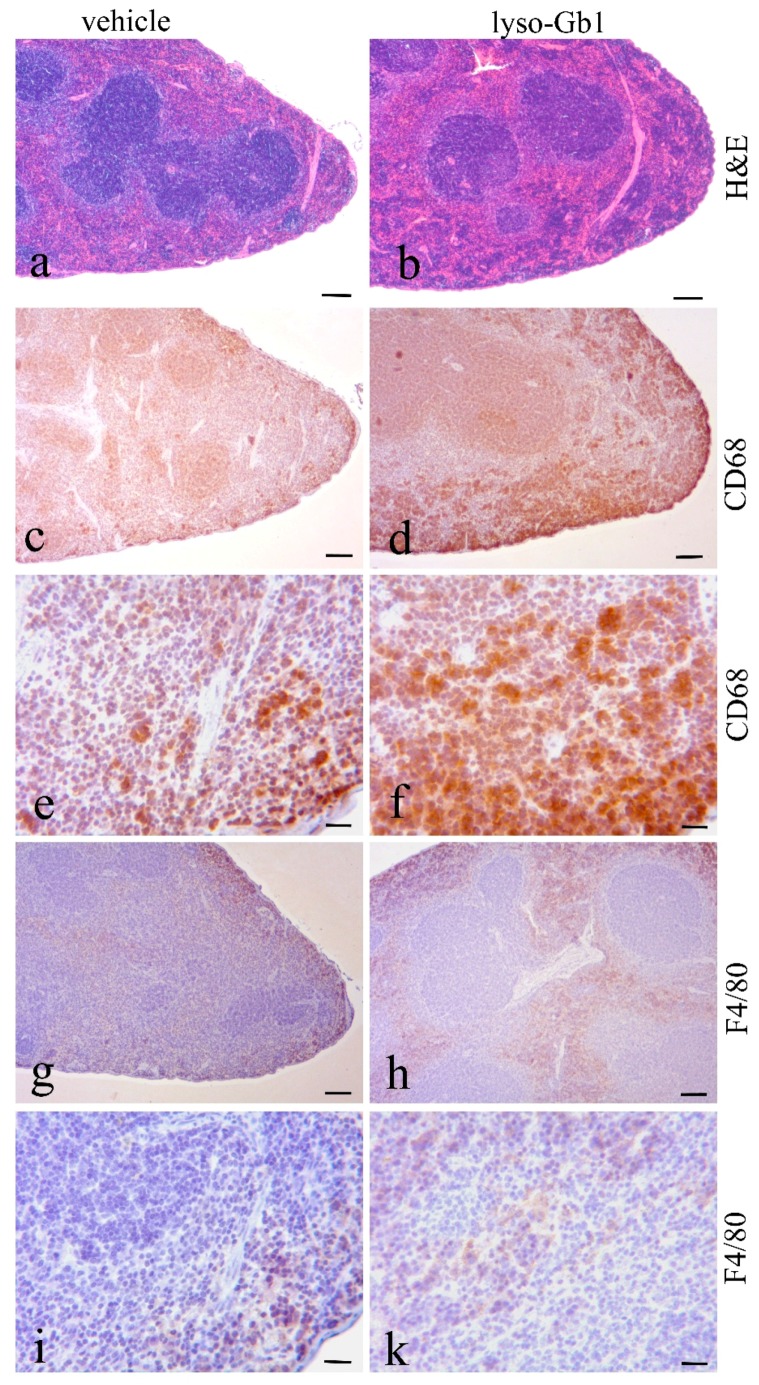
Increased number of CD68- and F4/80-positive cells in lyso-Gb1 treated mice spleen. Hematoxylin/eosin (H&E)-stained spleen sections from vehicle- and lyso-Gb1-treated mice showed similar splenic architecture with white and red pulp (**a**,**b**); Paraffin sections from the spleen of vehicle and lyso-Gb1 treated mice were immunohistochemically analyzed with anti-CD68 (**c**,**d** and high magnification **e**,**f**) and anti-F4/80 (**g**,**h** and high magnification **i**,**k**). Section from lyso-Gb1 treated mice showed a marked increase of the number of CD68 and F4/80 positive cells in comparison to vehicle controls. Scale bars: (**a**–**d**,**g**–**h**): 100 µm; (**e**,**f**,**i,k**): 20 µm.

**Figure 6:**
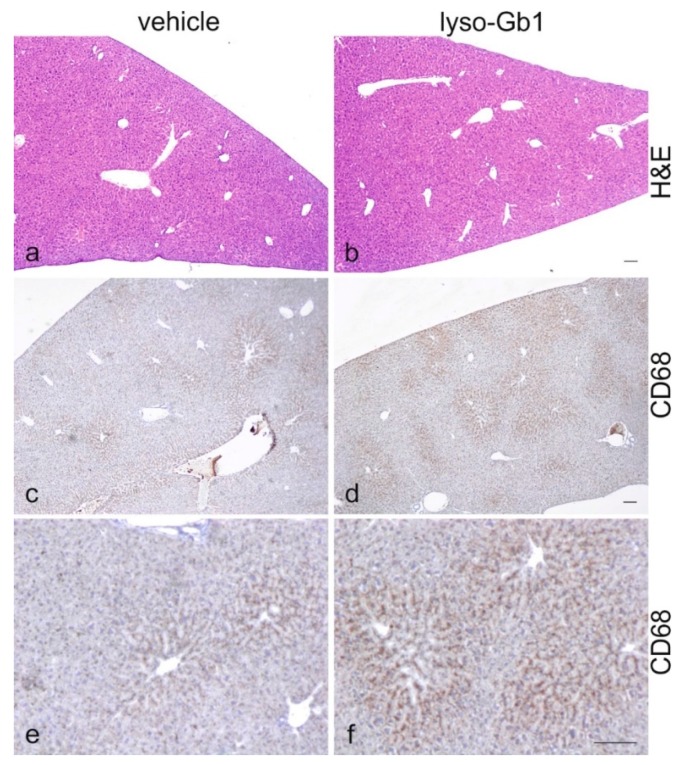
The moderately increased CD68-positive signal in the liver of lyso-Gb1 treated mice. H&E-stained liver sections from vehicle- and lyso-Gb1-treated mice showed a similar hepatic architecture (**a**,**b**). Paraffin sections from the liver of vehicle- and lyso-Gb1-treated mice were immunohistochemically analyzed with anti-CD68 (**c**,**d** and high magnification **e**,**f**). The sections of lyso-Gb1-treated mice showed mild alterations of CD68 immunoreactivity in comparison to vehicle controls. Scale bar 100 µm.

**Figure 7:**
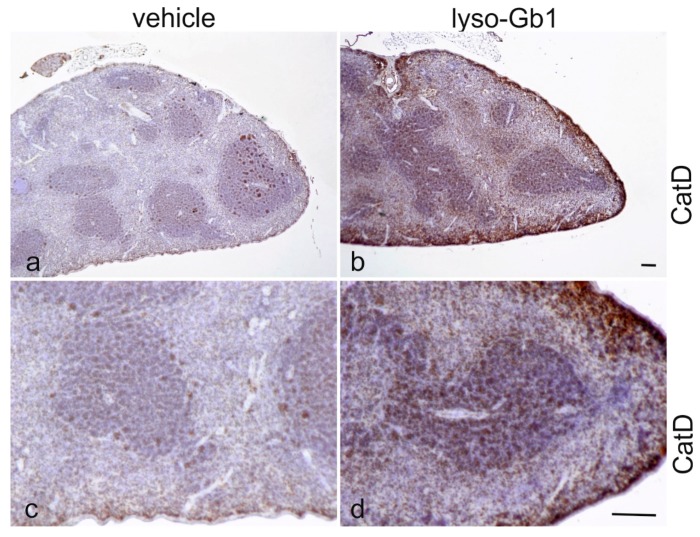
Increased number of cathepsin D positive cells in lyso-Gb1-treated mice spleen. Paraffin sections from the spleen of vehicle- and lyso-Gb1-treated mice were immunohistochemically analyzed with anti-cathepsin D (**a**,**b** and high magnification **c**,**d**). The section from the lyso-Gb1-treated mice showed a marked increase of the number of cathepsin D positive cells in comparison to vehicle controls. Scale bar 100 µm.

The impact of lyso-Gb1 treatment on spleen size was also examined, as anemia is often accompanied by splenomegaly, another hallmark of GD (Figure 4a,d). A significant, but moderate increase in spleen weight was observed in treated animals compared with controls, but no hepatomegaly was seen. To further investigate the molecular changes in these organs, Western blot analysis of macrophagic marker proteins CD68 and F4/80 was carried out to illustrate tissue inflammation. Consistent with organ weights, the levels of both proteins were elevated in the spleen, whereas milder changes were observed in the liver (Figure 4b,c). The increased cathepsin D level evidenced an altered cellular physiology arguing for a direct effect of lyso-Gb1 within the lysosomes of the cells (Figure 4b,c). A general histological stain of the spleen and liver using hematoxylin/eosin (H&E) did not reveal significant abnormalities between vehicle and lyso-Gb1 treated organs (Figure 5a,b and Figure 6a,b), but immunohistochemical analysis confirmed the Western blot results. In detail, H&E-stained sections of the spleen showed a similar morphology in white and red pulp in both vehicle and lyso-Gb1 treated mice (Figure 5a,b). Also, no change in liver histology was observed by H&E staining (Figure 6a,b). The immunohistochemical data revealed a strong increase of CD68 immunoreactivity in the spleen sections of lyso-Gb1 treated mice, when compared to vehicle controls (Figure 5c,d). In particular, an accumulation of CD68-positive cells was seen near the capsule (Figure 5e,f). CD68 immunoreactivity in the liver illustrated a moderate increase (Figure 6c–f) comparable to the Western blot results (Figure 4b,c). We further investigated F4/80 immunoreactivity in the spleen. Here, the signal intensity was increased in lyso-Gb1 treated mice compared to vehicle controls (Figure 5g,h). Higher magnification showed that the qualitative staining intensity of F4/80-positive cells was weak to medium compared to that of CD68- positive cells in the spleen of lyso-Gb1 treated mice (Figure 5e,f,i,k). The immunoreactivity of cathepsin D, a lysosomal marker, revealed an increased number of cathepsin D-positive cells in lyso-Gb1 treated mice compared to vehicle controls reviewed in the general stain (Figure 7a,b). Higher magnification of the sections indicated a stronger stain in the white and red pulp likewise (Figure 7c,d).

**Figure A1:**
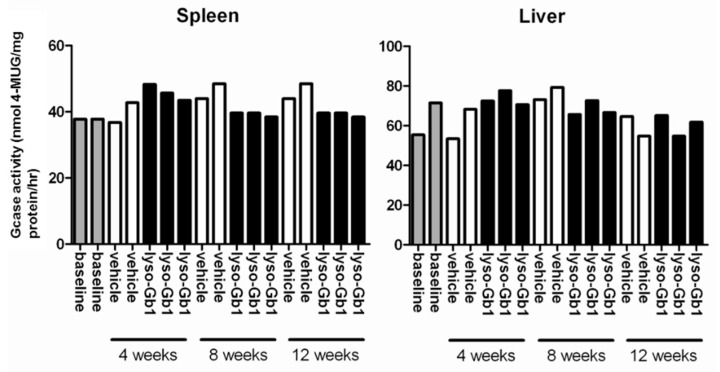
GCase activity in lyso-Gb1 treated mice spleen and liver. GCase activity measurement was carried out using artificial substare 4-MUG. The Spleen and liver GCase determination revealed that endogenous activity was not affected after four, eight and 12 weeks of treatment with lyso-Gb1, and that the enzyme remained unchanged over the three-month experimental period. Each sample was measured in two technical duplicates.

**Figure A2:**
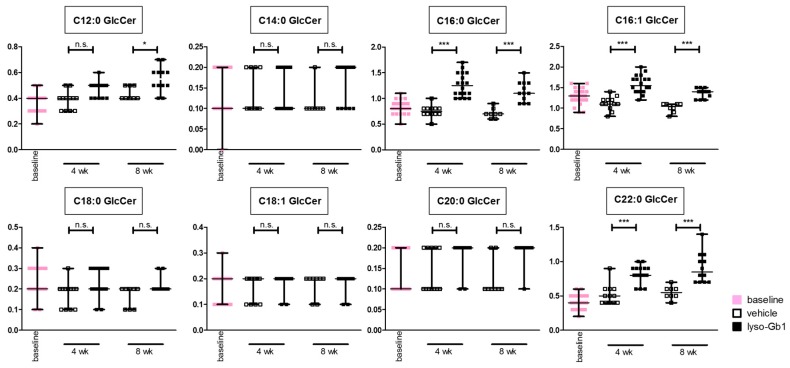
Glucosylceramide level in mouse blood. Glucosylceramide species with various fatty acid backbones were measured in the blood of four- and eight-week lyso-Gb1-treated mice. Small but significant elevations in glucosylceramide levels were observed for the C12:0, C16:0, C16:1 and C22:0 forms. The black, white and pink dots indicate the median and 25th and 75th percentiles, the whiskers of the graph show the minimum and maximum values. Differences between treatment groups at each time point were analyzed using the two-tailed Mann–Whitney test and considered statistically significant for *p* * < 0.05, *p* *** < 0.005, n.s.: not significant. Baseline (*n* = 34); vehicle, 4 weeks (*n* = 12); lyso-Gb1, 4 weeks (*n* = 18); vehicle, 8 weeks (*n* = 8); lyso-Gb1, 8 weeks (*n* = 12).

Lyso-Gb1 is a potential substrate of GCase, which poses the question of whether the treatment can influence proper enzyme function in the animals as assessed before (16). Enzyme measurements in liver and spleen lysates showed normal ex vivo activity (Figure A1) providing evidence that lyso-Gb1 did not significantly influence wild type GCase in the mice. This discovery is further supported by the fact that glucosylceramide levels are only moderately affected (Figure A2). It should provide a concise and precise description of the experimental results, their interpretation, as well as the experimental conclusions that can be drawn.

## 3. Discussion

Ten-week old C57BL/6JRj mice were treated with high subcutaneous doses (10 mg·kg^−1^ per day) of lyso-Gb1 for a period of up to 84 days (12 weeks). The mice developed a blood and splenic phenotype that resembles the phenotype observed in genetic mouse models of GD Type 1. The treatment led to an accumulation of lyso-Gb1 in all major tissues and an elevated blood lyso-Gb1 concentration of >500 ng/mL four weeks after the start of treatment, despite the presence of normal GCase. One explanation for this could be the lower capacity of GCase to hydrolyse lyso-Gb1 in comparison to glucosylceramide [16]. Typically, plasma lyso-Gb1 levels in GD patients range 50–250 ng/mL prior to enzyme replacement therapy [9], suggesting that the concentrations observed in the mouse circulation are comparable, taking into account the fact that, in humans, lyso-Gb1 blood levels exceed the plasma values by a factor of >2 [17]. It has been shown that lyso-Gb1 levels are associated with disease severity in GD patients [8,9]; it can therefore be expected that the observed lyso-Gb1 concentration, if initially causative to GD symptom onset, was high enough to produce a similar phenotype in mice as in patients, most typically hepatosplenomegaly, anemia and bone disease.

Glucosylceramide and glucosylsphingosine are believed to be responsible for macrophagic organ infiltration and the subsequent development of organomegaly. We therefore performed organ weight analysis of the treated mice. The observed increase in spleen lyso-Gb1 was accompanied with an increased spleen weight and an elevation of CD68 and F4/80 antigens confirmed by Western blot and immunohistochemistry. This could suggest either an early stage or chronic inflammation within this tissue due to an increased number of immune cells, likely macrophages [18,19], despite the variation in cell populations positively stained for CD68 and F4/80. In contrast, the heart, lung and kidneys appeared to be normal in size. Liver weight was slightly elevated at eight weeks of treatment, albeit not statistically significantly so, and the histological examination of liver sections using H&E staining suggested no pathophysiological condition. However, corresponding hepatic CD68 (Figure 4b,c and Figure 6c,f) and F4/80 (Figure 4b,c) levels appeared mildly elevated, and the marked enlargement of the liver was observed in a subsequent experiment where lyso-Gb1 treatment was started earlier (postnatal day 20) [20]. This experiment also confirmed the spleen enlargement and decreased Hb and Hct originally reported in genetic non-neuronopathic GD mice [21,22]. Notably, while a reduced platelet count is considered a hallmark symptom of GD, our examinations did not identify a decrease in the platelet count. In contrast, the platelet count appeared slightly elevated overall in both the original and follow-up study, although it was not statistically significant. No further progression in phenotype severity was observed after eight weeks of treatment with regards to organ weight and blood parameters; this suggests a physiological adaptation by the mice, or perhaps the absence of critical lyso-Gb1 concentrations at crucial target locations. Although we were able to demonstrate lysosomal activation by cathepsin D upregulation in the spleen, the efficiency of cellular lyso-Gb1 uptake remains unclear. Likely, its distribution does not completely reflect the mainly endolysosomal localization observed in GD patients and the genetic mouse models. This has essential consequences for the interpretation of the results. Endolysosomal storage of glucosylceramide and glucosylsphingosine affects the functionality of the lysosomes, which have an important role in the elimination of eobiotic compounds and inflammatory protection [23]. The recruitment of additional dysfunctional immune cells such as macrophages to the site of inflammation can accelerate disease progression, because the macrophages and neutrophils can no longer exert their protective role. This simplified picture describes the devastating vicious circle, which is, however, interrupted in the C57BL/6JRj mice, which appear to have functional guardian cells. This might also explain the absence of Gaucher cells in the lyso-Gb1-treated mice.

The absence of phenotypic progression could also argue for the involvement of other lipids linked to GD development, such as glucocerebroside and its isoforms and other co-regulated lipids, possibly accumulating due to the deficiency in GCase. Ultimately, it must be concluded that the treatment regime reported not fully recapitulating the progressive nature of the disease.

During the study, the mice displayed no obvious health problems or functional constraints, and did not lose weight. This finding is contrary to the situation in a genetic mouse model with no apparent central nervous system (CNS) involvement, which demonstrated a weight loss of 15% compared with healthy mice at 50 days of age, but no further progressive decline [24]. In this model, Mizukami and colleagues reported minimal glucosylceramide storage, and an absence of classic Gaucher cell tissue infiltration. However, their mice did display multisystemic inflammation reflecting elevated hepatic TNF-α and IL-1β expression, highlighting that rather inflammation is the key feature of GD that might not necessarily be typified by the presence of Gaucher cells. Our lyso-Gb1-treated mice showed a slight elevation of TNF-α and IL-1β in the blood, as observed by a multiplex immunoassay, possibly indicating B-cell proliferation. However, we did not find significant upregulation of most investigated cytokines (Table S1).

The maturity of the mice in this study (10-week old animals) may partially explain why the observed physiological changes in peripheral tissues did not display the entire phenotypic spectrum of GD symptoms. Earlier reports have indicated elevated lyso-Gb1 in the embryonic mouse [25], although this finding was with a neuronopathic GD mouse model. To date, there is no pre-natal study of lyso-Gb1 in a non-neuronopathic mouse model, but it is likely that lyso-Gb1 is also elevated during early developmental stages in GD type 1, as it has been shown to be elevated in tissue from very young GD patients [7].

In contrast to the GD patients and also the genetic mouse model of GD examined by Orvisky and colleagues [25], where the highest lyso-Gb1 levels were observed in the spleen, we observed a different distribution, with the highest lyso-Gb1 levels measured in the kidney and liver. The reported finding from Orvisky and colleagues would indicate that lyso-Gb1 in GD originates from intra-splenic macrophage sources, whereas in our model, the kidney and liver, not surprisingly, appeared to take up more of the subcutaneously administered lyso-Gb1. The negligible increase in lyso-Gb1 levels in the animal brain indicates that lyso-Gb1 is not able to cross the blood brain barrier. No CNS measurements were performed, as the animals displayed no behavioral abnormalities. Interestingly, the basal lyso-Gb1 level in the brain was higher than in the peripheral organs. This could argue for a good tolerance for lyso-Gb1 in the CNS. Altogether, the lyso-Gb1 triggered numerous significant molecular abnormalities known from the GD phenotype, but it was comparatively well-tolerated by the mice in terms of severe organ damage and mortality within the three-month period.

Most known GD models have the disadvantage of rapid decline, which hampers the close examination of disease progression. Most GD patients carry the N370S allele, which is associated with a late disease onset, mild symptoms and slow disease progression. It was shown that certain, primarily homozygous, patients remain asymptomatic throughout life [26]. Investigating the relationship between the pathophysiology of GD and the phenotype in the lyso-Gb1 treated animals can be used to gain a better understanding of common and milder GD alleles. The N370S mutation can, however, also be found in patients with an early disease onset [26]. Recent findings strongly suggest a critical role for the genetic background [27] in the phenotypic severity of GD animal models and patients. It can also be speculated that differences in the metabolic pathways of the animals contribute to disease development. Consequently, neither GD model does adequately reflect the complex spectrum of phenotypes manifested in human GD. Therefore, the strategy to subcutaneously treat C57BL/6JRj mice with lyso-Gb1 introduces another approach to unveil the intricate pathophysiological mechanism of GD.

The analysis of anti-inflammatory drug effects on the visceral signs of GD could help to reveal whether the observed inflammatory tissue reaction is sufficient to protect the mice from emerging disease signs, explaining their unaltered overall health status.

## 4. Materials and Methods

### 4.1. Animal Housing and Treatment

All animal experimental procedures were performed at Pharmacelsus GmbH (Saarbrücken, Germany) and were approved by, and conducted in accordance with the regulations of the local Animal Welfare Authorities (Licence No. C1-2.4.2./24-2015 at Landesamt für Gesundheit und Verbraucherschutz, Abteilung Lebensmittel und Veterinärwesen, Saarbrücken, Germany, registered on 07/07/2015, valid until 07/06/2018). Adult male C57BL/6JRj mice (10 weeks old, 23–26 g body weight at delivery, purchased from Janvier Labs, Le Genest-Saint-Isle, France) were housed in a temperature-controlled room (20–24 °C) and maintained in a 12 h light/12 h dark cycle. Food (ssniff^®^ R/M-H, 10 mm) and water were provided ad libitum.

Lyso-Gb1 (Matreya LLC, Pennsylvania State College, PA, USA) was administered by continuous subcutaneous infusion via ALZET osmotic mini pumps type 1004 delivering a daily dosage of 10 mg lyso-Gb1/kg body weight. The stock solution was prepared in DMSO:propylene glycol (1:1 ratio (*v/v*)) at 37 °C with ultrasonic bath treatment for 10 min. A concentration of 95 mg/mL lyso-Gb1 allowed a flow rate of 2.64 µL to achieve minimal vehicle infusion. The pumps were loaded with the dosing solution and primed prior to implantation. Implantation was carried out under isoflurane anesthesia according to the manufacturer’s instructions (Charles River Laboratories, Germany GmbH, Sulzfeld, Germany). The neck region was shaved and a small incision was made between the scapulae. Using a hemostat, a small pocket was formed by spreading the subcutaneous connective tissue apart. The pump was inserted into the pocket with the flow moderator pointing away from the incision. Finally, the incision was closed by two small sutures. This surgical procedure took 5–10 min. After recovery from anesthesia, the animals were transferred back to their home cage. The pumps were surgically replaced with fresh ones in each mouse every four weeks, to achieve lyso-Gb1 exposure over a total time period of 12 weeks. Animals were checked for clinical signs and mortality for two hours after pump implantation, and once daily throughout the experimental period. Body weight was documented once a week. The pumps were well tolerated and no mortalities were observed. Blood samples were collected in lithium-heparin sample tubes, using blood obtained from the lateral tail vein. At each sampling time point, two 20 µL aliquots were transferred to dried blood spot (DBS) filter cards (Centogene AG, Rostock, Germany). After the last sampling time point the mice were sacrificed by inhalation of an overdose of isoflurane, and organs were removed, frozen and stored for future examination. Where applicable, organs were intersected: half of the organ was immediately frozen in liquid nitrogen; the other half was formalin-fixed and paraffin-embedded for histological analysis (HISTALIM, Montpellier, France).

### 4.2. Sample Preparation from Dried Blood Spots for Liquid Chromatography (LC)-Multiple Reaction Monitoring Mass Spectrometry (MRM-MS)

Three punches of 3.2 mm in diameter were cut using a DBS puncher (Perkin Elmer LAS, Rodga, Germany) and placed in a 2.2 mL round-bottomed tube (Eppendorf, Hamburg, Germany). Fifty microliters of extraction solution (dimethylsulfoxid: water, 1:1) and 100 µL of internal standard solution (200 ng/mL lyso-Gb2 in ethanol; Matreya LLC, Pennsylvania State College) were added on top of the paper punches. Samples were mixed for 30 s and placed in an incubator (Heidolph, Schwabach, Germany) for 30 min at 37 °C under agitation at 700 rpm. After incubation, the tubes were sonicated for one minute at maximum power, and the resulting solution was transferred to an AcroPrep Filter Plate with a polytretrafluoroethylene membrane (PALL, Crailsheim, Germany) placed on a 96-well V-shape bottom plate (VWR, Darmstadt, Germany). The samples were filtered by centrifugation for 5 min at 3500 rpm in a Hermle Z300 plate centrifuge (Hermle Labortechnik, Wehingen, Germany) to remove any solid particles from the solution.

### 4.3. Preparation of Urine Samples for LC/MRM-MS Analysis

A total of 25 µL urine aliquots were added to 100 µL of internal standard (lyso-Gb2, 200 ng/mL) and 250 µL ethanol. The samples were cooled to 4 °C for 1 h to precipitate the urine proteins, after which they were spun in a benchtop centrifuge for 3 min at 14,500 rpm. The volume of each sample was quantitatively transferred to a 96-well filter plate, and further processed as described above.

### 4.4. Sample Preparation from Animal Organs for LC/MRM-MS

After extraction from the animal, organ samples for LC/MRM-MS were immediately deep-frozen in liquid nitrogen. The samples were lyophilized (Alpha 2-4 LSC, Christ, Osterode am Harz, Germany) and powdered using a pestle and mortar. Fractions of 2–5 mg powder were aliquoted into round-bottomed tubes. On top of the powder, 50 µL of extraction solution per mg powder was added and the sample was incubated for 5 min at 37 °C with agitation. Subsequently, the samples were frozen in liquid nitrogen for 30 s and sonicated using a boost function for 5 min. These incubation, freezing and sonication steps were repeated six times.

The resulting mixtures were vortexed, and 25 µL aliquots of the suspension were used for the lyso-Gb1 determination. Each sample was supplemented with 100 µL of internal standard (lyso-Gb2, 200 ng/mL) and 250 µL ethanol. The sample mixes were then cooled to 4 °C for 1 h to precipitate the membrane proteins, after which they were spun in a benchtop centrifuge for 3 min at 14,500 rpm. The volume of each sample was quantitatively transferred to a 96-well filter plate, and further processed as described above.

### 4.5. LC/MRM-MS Measurements

LC/MRM-MS analyses of lyso-Gb1 for both DBS and organ extracts were performed using a Waters Acquity ultraperformance liquid chromatography (UPLC) system (Waters, Wilmslow, UK) coupled with an ABSciex 5500 TripleQuad mass spectrometer (ABSciex, Darmstadt, Germany). Chromatographic runs were performed on a C8 column with a pore size of 3 mm (ACE columns, Mainz, Germany) using a flow rate of 0.9 mL/min preheated to 60 °C. The 10 µL extract was injected onto the column and the compounds were eluted using a linear gradient from 40% A (50 mM formic acid in water) to 100% B (50 mM formic acid in acetone:acetonitrile, 1:1 by volume). A 3:1 flow splitter was added upstream from the UPLC. The following MRM transitions were monitored: 624.3 → 282.2 for the internal standard (with a declustering potential of 30 V, collision energy of 38 V and collision cell exit potential of 10 V) and 462.3 → 282.2 for lyso-Gb1 (with a declustering potential of 28 V, collision energy of 30 V and collision cell exit potential of 10 V). MRM-MS analyses were performed in positive ion mode using the following parameters: curtain gas 40 psi, ion source voltage 5.5 kV, collision gas 8 psi, cone temperature 500 °C, source gas 1.45 psi, Source gas 2.60 psi, and entrance potential 10 V. For all batches analyzed, a standard curve was measured using seven dilutions of lyso-Gb1 in ethanol (concentrations in ng/mL: 0; 5; 10; 50; 100; 200; 1000).

### 4.6. Blood Analysis

Mice were exsanguinated via the retrobulbar venous plexus under isoflurane anesthesia before being sacrificed by overdose inhalation. EDTA samples of 200 µL whole blood were drawn from each animal to analyze aspartate aminotransferase (AST/GOT), leukocyte count, erythrocyte count, hemoglobin (Hb), hematocrit (PCV = packed cell volume, Hct), mean cell volume (MCV), mean cell Hb (MCH), mean cell Hb concentration. (MCHC) and platelet count (PLT) (IDEXX Bioresearch, Ludwigsburg, Germany).

### 4.7. Cytokine Analysis

The ProcartaPlex^®^ Multiplex Immunoassay (ebioscience, San Diego, CA, USA) was performed according to the manufacturer’s instructions. Differences between the animal groups were evaluated using automated MAGPIX analysis software that included quality control criteria.

### 4.8. Western Blotting

Liver and spleen samples were homogenized in radioimmunoprecipitation assay buffer containing proteinase and phosphatase inhibitors (Roche, Mannheim, Germany). Lysates were centrifuged at 15,000× *g*, 4 °C for 15 min to remove insoluble material, and the supernatant was collected. The protein concentration was measured using a Pierce Bicinchoninic Acid Protein Assay Kit (Thermo Fisher Scientific, Waltham, MA, USA). Typically, 100 µg of protein was loaded for electrophoresis on a 4–15% precast Tris-glycine gradient gel (BioRad, Munich, Germany). The protein was subsequently transferred to a nitrocellulose membrane using semi-dry transfer apparatus (BioRad, Munich, Germany) for immunodetection analysis. The target proteins were detected by rat anti-mouse F4/80 Cl:A3-1 (1:500, Biolegend, San Diego CA, USA), rat anti-CD68 clone FA-11 (1:200, Bio-Rad Laboratories, Raleigh, NC, USA), anti-cathepsin D antibody clone EPR3057Y (1:2,000 Abcam, Cambridge, UK), and mouse anti-GAPDH clone 6C5 (1:10,000, Abcam), each in tris-buffered saline and tween supplemented with 5% skimmed milk powder (Sigma Aldrich, Munich, Germany). Fluorescent conjugate secondary antibodies were applied for the detection with a Li-Cor Odyssey imaging system (Bad Homburg, Germany).

### 4.9. Histopathological Evaluation

After removal, the organs were fixed in formalin for 24 h and transferred into 70% ethanol. To ensure a non-biased comparison, the same parts of the organs were analyzed by HISTALIM (Montpellier, France). The samples were processed on the Peloris automaton (Leica, Wetzlar, Germany) according to the 4 h program validated for mouse organs. The samples were embedded in paraffin wax according to HISTALIM procedures. For the liver and spleen samples, a section (3–5 μm thickness) was prepared and deposited preferentially on Superfrost + slide (to ensure tissue adhesion) to be stained according to a validated hematoxylin/eosin (H&E) protocol. All the slides were digitalized with the Nanozoomer scanner (Hamamatsu Photonics, Hamamatsu, Japan) in bright field, with the objective ×20, without Z stack.

### 4.10. Immunohistochemistry

For the immunohistochemistry, 4–5 µm paraffin sections of the organs of eight-week treated mice were subjected to rat anti-CD68 (1:100), rat anti-mouse F4/80 (1:100), and anti-cathepsin D antibody (1:1000). Sections were then de-paraffinised, rehydrated and pre-treated in the microwaves in 0.1 M citrate buffer (5 min 850 W and 5 min 340 W) followed by consecutive incubation with 3% H_2_O_2_ in phosphate-buffered saline (PBS) to block endogenous peroxidases for 30 min, then 3% bovine serum albumin with 1.5% normal goat serum (NGS) in PBS for 1 h to block nonspecific epitopes. Subsequently, sections were exposed to the primary antibody in 3% NGS/PBS overnight at 4 °C. Depending on the primary antibody and after washing in PBS, the sections were sequentially incubated for 1 h with the secondary anti-rat, anti-mouse IgG, and anti-rabbit IgG (1:200; Vector, Burlingame, CA, USA), streptavidin-biotin-complex (ABC) reagent for 1 h (Vectastain-Elite; Vector, Burlingame) and then finally visualized with 3,-3,-diaminobenzidine (Sigma Aldrich), which was activated with H_2_O_2_. Sections were counterstained with hematoxylin, dehydrated, mounted with DePeX and coverslipped. The sections were imaged using a Keyence Biozero fluorescence microscope (Keyence GmbH, Neu-Isenburg, Germany) and a Nikon Optiphot-2 (NIKON GmbH, Düsseldorf, Germany). Digital data was processed with the Biozero observation application software from Keyence and Adobe Photoshop CS5 (Adobe Systems, Inc., San Jose, CA, USA).

### 4.11. Glucocerebrosidase Enzyme Activity Measurement

Fresh-frozen liver and spleen samples were homogenized in pH 4.5 adjusted ice-cold potassium-phosphate buffer (100 µL/mg tissue) supplemented with 0.15% Triton X-100 and 0.125% sodium taurocholate. The tissue suspension was forced 10 times through a 22-gauge needle, equipped with a 2 mL syringe to release cell association. Thereafter, the suspension was subjected to five freeze/thaw cycles followed by centrifugation at 15,000× *g* for 15 min at 4 °C to obtain a cleared lysate. The protein concentration of the GCase-containing extracts was measured and 9 µg whole protein was used for the enzymatic reaction using 2 mM final concentration 4-Methylumbelliferyl-β-d-glucopyranoside (4-MUG) as substrate. The reaction was terminated by the addition of 0.2 mL of 1.0 M glycine buffer (pH 10.5). The free fluorophore 4-MU was determined in a microplate reader (Tecan, Männedorf, Switzerland).

### 4.12. Data Analysis

Visualization and statistical data evaluation were carried out using GraphPad Prism 5 (GraphPad Software, Inc., La Jolla, CA, USA). Results are presented as median and range. The non-parametric two-tailed Mann–Whitney test was used to identify differences between treatment groups: hemoglobin, Hct and liver/spleen weight of control versus lyso-Gb1 treated mice at each indicated time point. Results were considered to be statistically significant for *p* * < 0.05, *p* ** < 0.01.

## 5. Conclusions

A three-month period of systemic subcutaneous administration of lyso-Gb1 to 10-week old male C57BL/6JRj mice caused mild anemia indicated by decreased Hb/Hct levels. No significant abnormalities in platelet or white blood cell counts were observed, indicating no acute effect on the bone marrow, which was supported by standard histological examination (not shown). The organs were apparently unable to degrade lyso-Gb1 at the high administration rate, and the stored lyso-Gb1 led to the up-regulation of the tissue inflammatory markers CD68 and F4/80 in the liver and spleen, as well as an increase in spleen weight. Plasma levels of the cytokine TNFα were elevated at four, 8 and 12 weeks after the start of treatment. Taken together, these findings demonstrate that lyso-Gb1 replicates a number of the molecular characteristics of a GD type 1, demonstrating its usefulness for further studies as a toxicological disease model.

## Figures and Tables

Figure abbreviations: GlcCer (Glucosylceramide); wk (weeks).

## Supplementary Materials

Supplementary materials can be found at www.mdpi.com/1422-0067/18/10/2192/s1.

## Abbreviations

4-MUG	4-Methylumbelliferyl-β- d-glucopyranoside
DBS	dried blood spot
GCase	Glucocerebrosidase
GD	Gaucher Disease
Hb	hemoglobin
Hct (PCV)	hematocrit
H&E	Hematoxylin and Eosin
Lyso-Gb1	glucosylsphingosine
LC/MRM-MS	liquid chromatography-multiple reaction monitoring mass spectrometry
MCH	mean cell Hb
MCHC	mean cell Hb concentration
MCV	mean cell volume
PLT	platelet count
