# Supplementary material for: Glucosylsphingosine Causes Hematological and Visceral Changes in Mice—Evidence for a Pathophysiological Role in Gaucher Disease
Source: Int J Mol Sci. 2017 Oct 20;18(10):2192. doi: 10.3390/ijms18102192 (PMC5666873; doi:10.3390/ijms18102192)
Supplement: Supplementary file 1 [file ijms-18-02192-s001.pdf]

Table S1

A

Median	body weight on day of analysis	AST (GOT)	Leuko-cytes	Erythro-cytes	Hb	PCV	MCV	MCH	MCHC	Thrombo-cytes	relative liver weight	relative spleen weight
	g	U/l	G/l	T/l	g/dl	%	fl	pg	g/dl	G/l	g/100g b.w.	g/kg b.w.
baseline	25.6	66.0	6.4	8.6	12.9	39.0	45.0	14.9	33.0	869.5	n.d.	n.d.
4 wk vehicle	28.7	56.0	7.7	9.0	13.2	40.5	45.5	14.9	32.0	896.0	n.d.	n.d.
4 wk lysogb1	27.3	47.0	8.2	8.5	12.3	37.5	44.0	14.4	33.0	1046.0	n.d.	n.d.
8 wk vehicle	30.2	43.5	9.6	9.3	13.5	41.5	44.0	14.6	33.0	1065.0	4.8	2.5
8 wk lysogb1	29.7	55.5	8.4	8.6	12.4	37.5	43.5	14.7	33.0	1226.0	5.3	3.3
12 wk vehicle	32.7	68.0	4.8	8.6	12.7	37.0	44.0	15.0	35.0	1268.0	5.0	2.4
12 wk lysogb1	32.5	75.0	5.8	8.3	12.2	36.0	43.0	14.6	34.0	1157.0	5.2	2.9

B

Median	GM-CSF	IFN-gamma	IL-1 beta	IL-10	IL-12p70	IL-13	IL-17A	IL-18	IL-2	IL-22	IL-23	IL-27	IL-4	IL-5	IL-6	IL-9	TNF-alpha
	pg/ml	pg/ml	pg/ml	pg/ml	pg/ml	pg/ml	pg/ml	pg/ml	pg/ml	pg/ml	pg/ml	pg/ml	pg/ml	pg/ml	pg/ml	pg/ml	pg/ml
baseline	0,00	0,85	0,46	0,00	0,83	0,00	7,14	109,82	1,30	37,73	9,28	9,66	1,35	6,15	0,59	0,00	0,00
4wk vehicle	0,00	0,27	0,23	0,00	0,45	0,00	3,43	100,23	0,00	10,23	4,78	5,78	0,81	5,25	3,31	0,00	0,00
4 wk lysogb1	4,41	0,85	0,46	0,00	1,21	0,45	4,22	136,52	1,30	24,71	5,40	6,56	1,42	7,02	16,85	0,00	2,58
8 wk vehicle	3,33	1,22	0,74	0,00	0,95	0,31	2,99	167,84	4,22	33,52	5,40	8,87	1,56	7,88	5,90	0,00	2,33
8 wk lysogb1	4,41	1,23	1,03	0,00	1,21	0,62	5,28	167,84	10,09	37,73	6,74	9,65	1,42	6,15	16,64	0,00	2,45
12 wk vehicle	3,33	0,66	0,07	0,00	0,95	0,15	2,64	142,83	0,00	24,33	4,01	9,66	1,57	4,78	7,06	0,00	0,08
12 wk lysogb1	5,20	1,41	1,29	0,00	1,34	0,31	4,95	173,61	0,00	24,91	20,63	9,27	2,08	7,02	20,71	0,00	1,20

Table S1. Extensive analysis of blood parameters and plasma cytokines. (**A**). Hb and Hct values appeared to be significantly changed after 4 and 8 weeks of treatment when compared to vehicle-treated control animals as well as baseline value. After 12 weeks no further regression of Hct values was observed. Leukocyte and thrombocyte counts and red-cell parameters with the exception of MCH appeared normal in the treated mice. However, thrombocytes showed a trend to elevate throughout the experimental phase compared to baseline, but not between groups. Spleen weights were moderately increased; (**B**). Cytokine analysis was carried out using ProcartaPlex® Multiplex Immunoassay. Median IL-1β and TNFα were elevated compared with vehicle-treated controls. *) Data have been tested for differences using Mann-Whitney test and deemed statistically significant (indicated in red) when $p < 0.05$ compared to control group and baseline value.
